# Pain experience and social support of endometriosis patients during the COVID-19 pandemic in Germany – results of a web-based cross-sectional survey

**DOI:** 10.1371/journal.pone.0256433

**Published:** 2021-08-25

**Authors:** Roxana Schwab, Katharina Anić, Kathrin Stewen, Mona W. Schmidt, Stefanie R. Kalb, Tanja Kottmann, Walburgis Brenner, Jana-Sophie Domidian, Slavomir Krajnak, Marco J. Battista, Annette Hasenburg

**Affiliations:** 1 Department of Obstetrics and Gynecology, University Medical Center of the Johannes Gutenberg University Mainz, Mainz, Germany; 2 CRO Dr. med. Kottmann, Hamm, Germany; International Medical University, MALAYSIA

## Abstract

**Background:**

Endometriosis is a chronic pain condition in premenopausal women. Pain is mainly characterized by pain intensity and may induce disability in all areas of daily life. Nevertheless, pain is influenced by emotional and social factors as well. Social distancing measures or quarantine, as reaction to rapidly rising infections with the COVID-19 virus due to the SARS-CoV-2 pandemic, were implemented across Europe to prevent the spread of the virus and social distancing measures were imposed by the German government by beginning of March 2020 with initiation of the lockdown by the end of March 2020. The objective of this study was to assess, how social distancing measures during the lockdown impacted the various aspects of pain perception in a group of chronic pain patients, such as women suffering from endometriosis.

**Methods:**

Between 6^th^ to 27^th^ April 2020, an online questionnaire was activated at internet platforms of endometriosis patients support groups. Participants were asked retrospectively at one time point about their visual pain intensity measured by the visual analogue scale (VAS) and pain disability via pain disability index (PDI) prior to initiation of social distancing measures in Germany (VAS_P_, PDI_P_), as well as the pain intensity and pain disability since implementation of social distancing measures (VAS_I_, PDI_I_). Differences of VAS and PDI previous and after implementation of social distancing measures were displayed as ΔVAS and ΔPDI. Pain experience and social support were assessed by a 5-point Likert scale.

**Results:**

285 participants completed at least one question regarding pain intensity, disability, pain experience or social support. Dysmenorrhea, the symptom with the highest level of pain assessed by VAS, decreased significantly during the SARS-CoV-2 pandemic compared to the time period prior to social isolation (45.30% respondents experienced improvemenet vs 40.50% who experienced worsening; p = 0.025). The global physical impairment improved significantly (improvement of pain induced disability in 48.20% vs 40.90% with worsening of pain symptoms; p = 0.032) after the implementation of social distancing measures. Pain experience was negatively affected by social distancing measures, since frequency of pain awareness increased in 43.6% (p<0.001) of participants and 30.0% (p<0.001) more participants experienced pain as a threat. Verbalization of pain experience was reduced in 36.6% (p = 0.001) of participants and 14.6% (p = 0.91), 21.9% (p<0.001) and 31.5% (p<0.001) of participants reported less social support from their partner, family and friends.

**Conclusions:**

Physical pain and disability on one hand and emotional and social pain experience on the other were differentially affected by the emerged emotional, social and health care constraints related to the SARS-CoV-2 pandemic.

## Introduction

Endometriosis is a chronic inflammatory disease defined by growth of endometrial-like tissue outside the uterine cavity [1, 2]. The etiology of endometriosis is not fully understood [1]. Since diagnosis requires surgical visualization with histological examination [2], the real prevalence of endometriosis is unknown, but it is estimated that 10% of women in the fertile life span and up to 50% of women who experience infertility suffer from endometriosis, which sums up to approximately 190 million women worldwide [1, 3–5].

Pain is one of the key clinical presentations in patients with endometriosis. Symptoms may vary in terms of pain localization, persistence, intensity and induced disability [6]. Women may experience cyclical or non-cyclical pelvic pain, such as dysmenorrhea, dyspareunia or dyschezia [2, 4, 7]. Pain experience is a multidimensional phenomenon and is an interplay of physical, psychological and social variables [8, 9]. Moreover, pain may lead to detrimental effects on Health-Related Quality of Life (HRQoL). HRQoL is defined on the basis of attributes valued by patients and subsume several aspects of life such as relationship with the partner, social life, physical and psychological well-being [10, 11]. Since endometriosis is a non-curable disease, the primary long-term goal of treatment is the improvement or the maintenance of HRQoL.

The COVID-19 coronavirus disease 2019 (COVID-19) pandemic is caused by infection with the severe acute respiratory syndrome coronavirus 2 (SARS-CoV-2) [12, 13]. The novel infectious disease was reported in China by the end of December 2019 and spread rapidly around the world within a couple of weeks. The World Health Organization declared the outbreak as a “Public Health Emergency of International Concern” on 01/30/2020 and as a pandemic on 03/11/2020 [12]. Considering the lack of causal therapy and the predominately airborne nature of the infection, public-health interventions such as isolation of infected persons and quarantine of contact persons as well as wide spread social distancing measures and lockdown to slow down and prevent the transmission of the virus were implemented by health care systems and governments around the world [12–14]. In Germany, wide spread social distancing measures were implemented by early March 2020 and even more strict measures, such as the lockdown, were implemented by the government by the end of March 2020 [12, 15]. As a result of infection control precautions, diversion of health care resources and fear of infection with the novel corona virus [16, 17], chronic pain patients faced the threat of deterioration of HRQoL due to delayed or inappropriate treatment [18–20].

The objectives of this study were the assessment of alterations in pain perception and specific pain induced disabilities, as well as changes in the emotional and social aspects of pain in endometriosis patients during social isolation or quarantine due to the COVID-19 pandemic.

## Methods

To assess the impact of the government-imposed social distancing or quarantine, an online questionnaire was developed. It contained 18 question blocks and took about 30 minutes to complete. Women were eligible for the study if they were older than 18 years, had a histologically confirmed history of endometriosis and agreed to participate. Data collection and analysis were performed anonymously. Recruitment was conducted via a direct link to the survey and an invitation to participate distributed via the internet platforms of patients support groups. The survey link was active from 6th to 27th April 2020, during the government-imposed social distancing or quarantine in Germany. A full copy of the questions which were considered for the present evaluation can be found in S1 File. The questionnaire covered demographic and pain related characteristics, as well as psychological variables. Pain intensity was assessed by the visual pain scale (VAS) and pain induced disability by the pain disability index (PDI). The VAS is a continuous scale ranging from 0 to 100 (100 being the strongest imaginable pain) [21]. The PDI assesses the degree of pain caused disability in seven areas of daily life (family/home responsibilities, recreation, social activity, occupation, sexual behavior, self-care, and life-support activity). Each item score ranges from 0 (no interference) to 10 (total interference). Thus, the total PDI score can range from 0 to 70 [22]. Participants were asked to answer the questionnaire once and to recall their pain intensity and pain disability over the past 4 weeks previous to the implementation of social distancing measures (VAS_P_, PDI_P_) as well as the pain intensity and pain disability since implementation of social distancing measures (VAS_I_, PDI_I_). Differences of pain intensity (ΔVAS) and pain disability (ΔPDI) were evaluated for the participants who answered the questionnaire for both time periods.

All other measures describing the specific situation since the beginning of social distancing or quarantine, such as details about perceived social support, as well as perceived pain management and use of medication, were assessed by an incremental 5-point Likert scale. For data analysis, the Likert scale variables “strongly disagree” and “disagree” on one hand and “agree” and “strongly agree” on the other were clustered.

### Statistical analysis

Data were analyzed using SPSS 24.0 (SPSS Inc., Chicago, IL, U.S.A). Descriptive statistics are presented as mean, standard deviation (±SD), median, interquartile range (IQR) (range between Q1 = 25^th^ and Q3 = 75^th^ percentile), or proportions (%), as appropriate. Assessments of normality were performed with the Kolmogorow-Smirnow Test (KM). An explorative comparison of the pain and the disability level (PDI score) for the assessment period of pre- and during isolation was performed by the Wilcoxon matched-pairs signed-rank test, as this method compares if two set of scores coming from the same participants are symmetrically distributed around zero. Changes for the variables assessed by Likert scale were assessed by chi-square (χ^2^) test.

For sensitivity analyses regarding pain intensity and pain induced disability, outliers lying outside three standard deviations were removed from the sample size. Additionally, we performed sensitivity analyses including only those respondents who answered all questions regarding pain intensity and those who answered all questions regarding pain induced disability, respectively.

Significance level was set at p≤0.05. As p-values were not adjusted for multiple testing, all results need to be interpreted in an exploratory manner.

### Ethics approval

This survey was approved by the Ethics Committee of the *Landesärztekammer Rheinland-Pfalz*, approval number 2020–14963 on 03^th^ of April 2020.

## Results

This article provides a descriptive analysis of the impact of social distancing, isolation or quarantine on pain intensity and pain induced disability, as well as changes in daily self-management behavior and relationships during lockdown. 413 participants met the inclusion criteria and accessed the questionnaire, but only 285 participants answered at least one question regarding pain intensity, pain induced disability, pain perception or social support. To understand the differences between those who did (group “Respondents”) or did not (group “None”) answer the questions, we assessed the demographic and clinical characteristics in both groups (Table 1). No significant statistical differences were detected regarding the demographic and clinical variables, except the duration of social isolation or quarantine, which was significantly longer (median 6 days longer) in the group of “Respondents”.

**Table 1:** Demographic and clinical characteristics of the total study sample showing differences between those participants who did not complete any question regarding pain intensity, pain induced disability, pain perception or social support (group “None”) versus those who completed at least one of the questions (group “Respondents”).

Variable		None	Respondents	p-value
		(N = 128)	(N = 285)	(group “None” vs group “Respondents”)
**Age** (years)	Mean (SD)	32.19 (7.00),	32.05 (7.07)	0.908^1^
	Median (IQR)	31.00 (27.00–37.50)	31.00 (27.00–36.00)	
**Having a stable partnership**				
Yes	% of N	71.30	77.90	0.191^2^
no	% of N	28.70	22.10	
**Time since social isolation or quarantine** (days)	Mean (SD)	24.60 (13.53)	27.75 (11.93)	**0.003** ^1^
	Median (IQR)	21.00 (20.00–30.00)	27.00 (21.00–32.00)	
**Reduction of social contacts**				
“Not at all” to “moderate”	% of N	31.90	27.40	0.397^2^
“Considerable” or “significantly”	% of N	68.10	72.60	
**Time since diagnosis of endometriosis** (years)	Mean (SD)	3.86 (4.62)	4.43 (4.83)	0.184^1^
	Median (IQR)	2.00 (1.00–4.00)	3.00 (1.00–5.00)	
**Age at diagnosis of endometriosis** (years)	Mean (SD)	28.25 (6.75)	27.63 (6.25)	0.276^1^
	Median (IQR)	29.00 (24.00–33.00)	27.00 (23.00–32.50)	
**Time since pain onset** (years)	Mean (SD)	13.15 (7.63)	14.02 (7.85)	0.337^1^
	Median (IQR)	12.00 (7.00–18.0)	13.00 (8.00–20.00)	
**Diagnostic delay of endometriosis** (years)	Mean (SD)	9.39 (6.99)	9.62 (6.87)	0.949^1^
	Median (IQR)	9.00 (5.00–18.00)	9.00 (4.50–14.00)	
**Pain quality**				
Continuous pain	% of N	34.10	35.40	0.817^2^
Pain peaks	% of N	65.90	64.60	

N = Number of women for which data were available, SD = standard deviation, IQR: Interquartile Range, vs = versus, Values in bold indicate statistical significance, as the level of statistical significance was set to p≤0.05 (^1^ = Mann-Whitney-U-test or ^2^ = χ^2^ -test)

**Fig 1:**
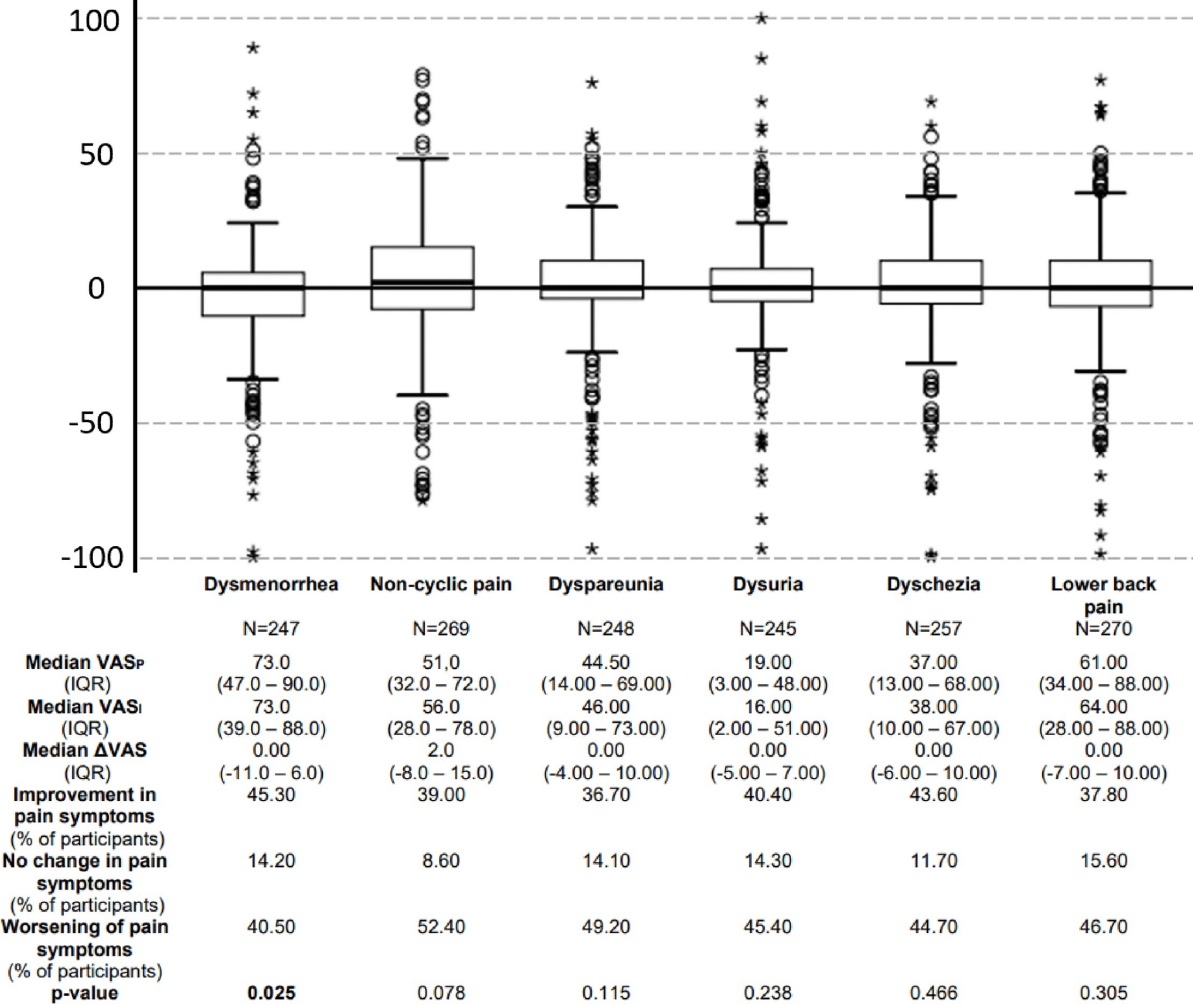
Differences of pain intensity (ΔVAS) during social isolation or quarantine (VAS_I_) and previous to social isolation or quarantine (VAS_P_). Boxplots of the differences of pain intensity are displayed in all assessed pain modalities. Positive values mean increased pain during social isolation or quarantine. Negative values mean decreased pain during social isolation or quarantine. The value 0 means no changes in pain intensity. Data are presented as median values (IQR) and percentage of respondents who experienced decreased, increased or no change in pain intensity. N represents the number of women who answered the question about pain intensity regarding both time periods: Before and after social isolation or quarantine. Values in bold indicate statistical significance (Wilcoxon test), as the level of statistical significance was set to p≤0.05.

Dysmenorrhea was the symptom with the highest pain intensity, followed by lower back pain, non-cyclic pain, dyspareunia, dyschezia and dysuria (Fig 1). Pain intensity decreased significantly for dysmenorrhea (median ΔVAS (IQR): 0.00 (-11.0–6.0); p = 0.025) during isolation or quarantine compared to the pain intensity before isolation or quarantine but remained unchanged for all other pain modalities (all p>0.05) (Fig 1).

Sensitivity analyses for pain intensity were carried out by repeating the Wilcoxon matched-pairs signed-rank tests after removing the outliers from the “Respondents” sample group for each assessed pain intensity variable in particular (Table 2).

**Table 2:** Sensitivity analyses of differences of pain intensity (ΔVAS) during social isolation or quarantine (VAS_I_) and previous to social isolation or quarantine (VAS_P_) after removing the outliers.

	Dysmenorrhea	Non-cyclic pain	Dyspareunia	Dysuria	Dyschezia	Lower back pain
	N = 241	N = 265	N = 242	N = 238	N = 250	N = 265
**Median VAS**_**P**_	73.00	51.00	43.50	19.00	36.00	61.00
(IQR)	(48.00–90.00)	(32.00–72.00)	(14.00–68.00)	(3.00–46.00)	(12.00–66.00)	(34.00–87.00)
**Median VAS**_**I**_	73.00	56.00	47.00	16.00	41.00	65.00
(IQR)	(41.00–87.00)	(29.00–78.00)	(9.00–73.00)	(2.00–51.00)	(10.00–67.00)	(29.00–89.00)
**Median ΔVAS**	0.00	2.00	0.50	0.00	0.00	0.00
(IQR)	(-10.00–5.00)	(-8.00–15.00)	(-4.00–10.00)	(-4.00–7.00)	(-5.00–11.00)	(-7.00–10.00)
**Improvement in pain symptoms**	45.25	38.50	35.50	39.90	42.40	37.00
(% of participants)						
**No change in pain symptoms**	14.50	8.70	14.50	14.70	12.00	15.80
(% of participants)						
**Worsening of pain symptoms**	40.25	52.80	50.00	25.49	45.60	47.20
(% of participants)						
**p-value**	**0.020**	**0.042**	**0.033**	0.172	0.174	0.157

Data are presented as median values (IQR) in pain intensity previous (VAS_P_) and during social isolation or quarantine (VAS_I_) and as differences of pain intensity (ΔVAS), as well as percentage of respondents who experienced improvement, worsening or no change in pain intensity. Positive values (ΔVAS), mean increased pain during social isolation or quarantine. Negative values (ΔVAS), mean decreased pain during social isolation or quarantine. The value 0 (ΔVAS), means no changes in pain intensity. N represents the number of women who answered the question about pain intensity regarding both time periods: Before and after social isolation or quarantine. Values in bold indicate statistical significance (Wilcoxon test), as the level of statistical significance was set to p≤0.05.

Additionally, sensitivity analyses were carried out by repeating the Wilcoxon matched-pairs signed-rank tests for those who completed all variables regarding the pain intensity (N = 201) (Table 3). The pattern of results in both sensitivity analyses regarding pain intensity confirmed the main findings in the primary Wilcoxon tests.

**Table 3:** Sensitivity analyses of differences of pain intensity (ΔVAS) during social isolation or quarantine (VAS_I_) and previous to social isolation or quarantine (VAS_P_) in the group of those respondents who completed all questions regarding pain intensity.

	Dysmenorrhea	Non-cyclic pain	Dyspareunia	Dysuria	Dyschezia	Lower back pain
	N = 201	N = 201	N = 201	N = 201	N = 201	N = 201
**Median VAS**_**P**_	73.0	51,00	44.00	16.00	32.00	59.00
(IQR)	(47.00–90.00)	(29.00–72.00)	(11.00–69.00)	(2.00–45.00)	(11.00–66.00)	(34.00–87.00)
**Median VAS**_**I**_	73.0	55.00	43.00	14.00	34.00	63.00
(IQR)	(37.00–88.00)	(25.00–78.00)	(7.00–73.00)	(2.00–49.00)	(8.00–65.00)	(28.00–88.00)
**Median ΔVAS**	0.00	2.0	0.00	0.00	0.00	0.00
(IQR)	(-10.00–5.00)	(-9.0–15.00)	(-4.00–8.00)	(-4.00–6.00)	(-5.00–10.00)	(-8.00–11.00)
**Improvement in pain symptoms**	44.80	38.80	35.80	39.80	41.80	38.30
(% of participants)						
**No change in pain symptoms**	16.40	8.50	19.90	15.90	13.40	16.90
(% of participants)						
**Worsening of pain symptoms**	38.80	52.70	48.30	44.30	44.80	44.80
(% of participants)						
**p-value**	0.051	0.112	0.195	0.357	0.233	0.370

Data are presented as median values (IQR) in pain intensity previous (VAS_P_) and during social isolation or quarantine (VAS_I_) and as differences of pain intensity (ΔVAS), as well as percentage of respondents who experienced improvement, worsening or no change in pain intensity. Positive values (ΔVAS), mean increased pain during social isolation or quarantine. Negative values (ΔVAS), mean decreased pain during social isolation or quarantine. The value 0 (ΔVAS), means no changes in pain intensity. N represents the number of women who answered the question about pain intensity regarding both time periods: Before and after social isolation or quarantine. The level of statistical significance (Wilcoxon test) was set to p≤0.05.

**Fig 2:**
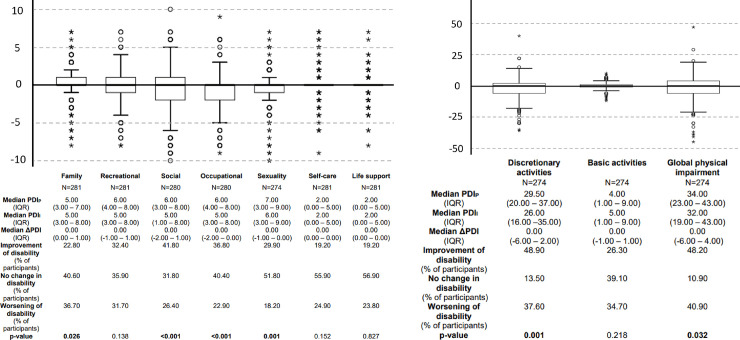
Differences of pain disability index (ΔPDI) during social isolation or quarantine (PDI_I_) and previous to social isolation or quarantine (PDI_P_). **(A)** Boxplots of the difference of pain disability index during social isolation or quarantine (PDI_I_) and previous to social isolation or quarantine (PDI_P_) are displayed in all assessed areas of life (“family”, “recreational”, “social”, “occupational”, “sexuality”, “self-care” and “life support”). **(B)** Boxplots of the difference of pain disability index during social isolation or quarantine (PDI_I_) and previous to social isolation or quarantine (PDI_P_) are displayed in the sum scores discretional activities (sum score of: “family”, “recreational”, “social”, “occupational” and “sexuality”), the sum score basic activities (sum score of: “self-care” and “life support”), and the global physical impairment as the sum score of all assessed areas of daily life. Positive values mean increased pain disability during social isolation or quarantine. Negative values mean decreased pain disability during social isolation or quarantine. The value 0 means no changes in pain disability. Data are presented as median values (IQR) and percentage of respondents who experienced decreased, increased or no change in pain induced disability. N represents the number of women who answered the questions about pain disability regarding both time periods: Previous and after isolation or quarantine. Values in bold indicate statistical significance (Wilcoxon test), as the level of statistical significance was set to p≤0.05.

The PDI questionnaire was used to assess the change of functional disability caused by pain during social isolation with respect to the period prior to isolation. Significant improvement of function occurred in the areas of social (median ΔPDI (IQR): 0.00 (-2.0–1.0); p<0.001), occupational (median ΔPDI (IQR): 0.00 (-2.0–0.0); p<0.001) and sexual (median ΔPDI (IQR): 0.00 (-1.0–0.0); p = 0.001) functioning (Fig 2A). In contrast, deterioration of function was observed with respect to family functioning (median ΔPDI (IQR): 0.00 (0.0–1); p = 0.026) (Fig 2A). The global PDI score, a substitute for the global physical impairment, improved significantly during social isolation or quarantine (median ΔPDI (IQR): 0.0 (-6.0–4.0); p = 0.032) (Fig 2B).

Sensitivity analyses for pain induced disability were carried out by repeating the Wilcoxon matched-pairs signed-rank tests after removing the outliers from the “Respondents” sample group for each assessed pain induced disability variable in particular (Table 4).

**Table 4:** Sensitivity analyses of differences of pain disability index (ΔPDI) during social isolation or quarantine (PDI_I_) and previous to social isolation or quarantine (PDI_P_) after removing the outliers.

	Family	Recreational	Social	Occupational	Sexuality	Self-care	Life support	Discretionary activities	Basic activities	Global physical impairment
	N = 273	N = 277	N = 279	N = 278	N = 265	N = 270	N = 276	N = 268	N = 267	N = 268
**Median PDI**_**P**_	5.00	6.00	6.00	6.00	7.00	2.00	2.0	29.00	4.00	34.00
(IQR)	(3.00–7.00)	(4.00–8.00)	(3.00–8.00)	(4.00–8.00)	(3.00–9.00)	(0.00–5.00)	(0.0–5.0)	(20.00–36.00)	(1.00–9.00)	(23.00–42.50)
**Median PDI**_**I**_	5.00	5.00	5.00	5.00	6.00	2.00	2.0	26.50	5.00	32.00
(IQR)	(3.00–8.00)	(3.00–8.00)	(1.00–8.00)	(3.00–8.00)	(3.00–9.00)	(0.00–5.00)	(0.0–5.0)	(16.00–35.50)	(1.00–9.00)	(19.50–43.00)
**Median ΔPDI**	0.00	0.00	0.00	0.00	0.00	0.00	0.0	0.0	0.00	0.00
(IQR)	(0.00–1.00)	(-1.00–1.00)	(-2.00–1.00)	(-2.00–0.00)	(-1.00–0.00)	(0.00–0.00)	(0.0–0.0)	(-5.00–2.00)	(-1.00–1.00)	(-5.00–4.00)
**Improvement of disability**	21.20	32.10	41.90	36.70	27.90	18.10	18.50	48.10	25.10	47.40
(% of participants)										
**No change in disability**	41.80	36.50	31.90	40.60	53.60	58.10	58.00	13.80	40.10	11.20
(% of participants)										
**Worsening of disability**	37.00	31.40	26.20	22.70	18.50	23.70	23.60	38.10	34.80	41.40
(% of participants)										
**p-value**	**0.003**	0.126	**<0.001**	**<0.001**	**0.009**	0.142	0.687	**0.004**	0.082	0.075

Data are presented as median values (IQR) of pain induced disability previous (PDI_P_) and during social isolation or quarantine (PDI_I_) and as differences of pain induced disability (ΔPDI), as well as percentage of respondents who experienced improvement, worsening or no change in pain induced disability. Positive values (ΔPDI), mean worsening of disability during social isolation or quarantine. Negative values (ΔPDI), mean improvement of disability during social isolation or quarantine. The value 0 (ΔPDI), means no changes in pain induced disability. N represents the number of women who answered the question about pain disability regarding both time periods: Before and after social isolation or quarantine. Values in bold indicate statistical significance (Wilcoxon test), as the level of statistical significance was set to p≤0.05.

Additionally, sensitivity analyses were carried out by repeating the Wilcoxon matched-pairs signed-rank tests for those who completed all variables regarding pain induced disability (N = 273) (Table 5). The pattern of results in both sensitivity analyses regarding pain induced disability confirmed the main findings in the primary Wilcoxon tests.

**Table 5:** Sensitivity analyses of differences of pain disability index (ΔPDI) during social isolation or quarantine (PDI_I_) and previous to social isolation or quarantine (PDI_P_) in the group of those respondents who completed all questions regarding pain induced disability.

	Family	Recreational	Social	Occupational	Sexuality	Self-care	Life support	Discretionary activities	Basic activities	Global physical impairment
	N = 273	N = 273	N = 273	N = 273	N = 273	N = 273	N = 273	N = 273	N = 273	N = 273
**Median PDI**_**P**_	5.00 (3.00–7.00)	6.00 (4.00–8.00)	6.00 (3.00–8.00)	6.00 (4.00–8.00)	7.00 (3.00–9.00)	2.00 (0.00–5.00)	2.00 (0.00–5.00)	30.00 (20.00–37.00)	4.00 (1.00–9.00)	34.00 (23.00–43.00)
(IQR)										
**Median PDI**_**I**_	5.00 (3.00–8.00)	5.00 (3.00–8.00)	5.00 (1.00–8.00)	5.00 (3.00–8.00)	6.00 (3.00–9.00)	2.00 (0.00–5.00)	2.00 (0.00–5.00)	26.00 (16.00–35.00)	5.00 (1.00–9.00)	32.00 (19.00–43.00)
(IQR)										
**Median ΔPDI**	0.00 (0.00–1.00)	0.00 (-1.00–1.00)	0.00 (-2.00–1.00)	0.00 (-2.00–0.00)	0.00 (-1.00–0.00)	0.00 (0.00–1.00)	0.00 (0.00–0.00)	0.00 (-6.00–2.00)	0.00 (-1.00–1.00)	0.00 (-6.00–4.00)
(IQR)										
**Improvement of disability**	22.70	32.60	41.80	37.00	29.70	19.80	19.00	48.70	26.40	48.00
(% of participants)										
**No change in disability**	40.70	35.50	31.50	40.30	52.00	54.90	57.10	13.60	38.80	11.00
(% of participants)										
**Worsening of disability**	36.6	31.90	26.70	22.70	18.30	25.30	23.90	37.70	34.80	41.00
(% of participants)										
**p-value**	**0.023**	0.157	**<0.001**	**<0.001**	**0.001**	0.163	0.831	**0.002**	0.218	**0.036**

Data are presented as median values (IQR) of pain induced disability previous (PDI_P_) and during social isolation or quarantine (PDI_I_) and as differences of pain induced disability (ΔPDI), as well as percentage of respondents who experienced improvement, worsening or no change in pain induced disability. Positive values (ΔPDI), mean worsening of disability during social isolation or quarantine. Negative values (ΔPDI), mean improvement of disability during social isolation or quarantine. The value 0 (ΔPDI), means no changes in pain induced disability. N represents the number of women who answered the question about pain disability regarding both time periods: Before and after social isolation or quarantine. Values in bold indicate statistical significance (Wilcoxon test), as the level of statistical significance was set to p≤0.05.

Pain cognition changed significantly during social isolation or quarantine. 43.6% (p<0.001) patients were significantly more frequent aware of pain and 29.3% (p = 0.02) more patients experienced pain as a disturbing event (Table 6). Nevertheless, 43.6% more participants had the possibility to relax more or significantly more despite the pain (Table 6). Verbalization of pain experience was reduced in 36.6% (p = 0.001) of participants (Table 6). 15.9% (p = 0.31) of participants increased the intake of over-the-counter pain medication and 15.9% (p = 0.91) increased the intake of prescription-only pain medication (Table 6).

**Table 6:** Alterations in pain perception and medication during social isolation or quarantine.

Variable	% of N	p-value
**Frequency of pain awareness/ pain hypervigilance**	(N = 280)	
Significantly less or less	14.7	
Not changed	41.7	**p<0.001**
More or significantly more	43.6	
**Experience of stress because of pain**	(N = 280)	
Significantly less or less	28.9	
Not changed	33.9	p = 0.09
More or significantly more	37.2	
**Possibility to relax despite pain**	(N = 280)	
Significantly less or less	40.0	
Not changed	28.6	p = 0.08
More or significantly more	31.4	
**Ability to cope with pain**	(N = 280)	
Significantly less or less	31.7	
Not changed	43.9	p = 0.09
More or significantly more	24.3	
**Experience of pain as bothersome/disturbing event**	(N = 280)	
Significantly less or less	19.6	
Not changed	51.1	**p = 0.02**
More or significantly more	29.3	
**Experience of pain as a threat**	(N = 280)	
Significantly less or less	16.1	
Not changed	53.9	**p<0.001**
More or significantly more	30.0	
**Verbalization of pain experience**	(N = 279	
Significantly less or less	36.6	
Not changed	41.5	**P = 0.001**
More or significantly more	21.9	
**Intake of over-the-counter pain medication**	(N = 278)	
Significantly less or less	19.4	
Not changed	64.7	p = 0.31
More or significantly more	15.9	
**Intake of prescription-only pain medication**	(N = 276)	
Significantly less or less	15.6	
Not changed	68.5	p = 0.91
More or significantly more	15.9	

N: Number of women for which data were available.

Values in bold indicate statistical significance, as the level of statistical significance was set to p≤0.05.

The perceived social support received from the partner remained unchanged (p = 0.91), while the social support received from family and friends was perceived as “less” or “significantly less” by 21.9% (p<0.001) and 31.5% (p<0.001) of endometriosis patients, respectively (Table 7). Additionally, the empathy from the partner towards the experienced pain was not significantly altered during the period of social isolation or quarantine, while the pain experienced by the participants was taken “less” or “considerable less” serious in 13.5% (p = 0.03) by the family and in 19.5% (p<0.001) by friends, respectively (Table 7).

**Table 7:** Alterations in social support regarding pain experience during social isolation.

Variable	Partner	Family	Friends
	% of N	% of N	% of N
**Support during pain experience by**	(N = 260)	(N = 274)	(N = 276)
Significantly less or less	14.6	21.9	31.5
Not changed	71.2	70.4	63.4
More or significantly more	14.2	7.7	5.1
p-value	p = 0.91	**p<0.001**	**p<0.001**
**Pain taken seriously by**	(N = 259)	(N = 274)	(N = 276)
Significantly less or less	10.4	13.5	19.5
Not changed	79.9	78.8	74.7
More significantly more	9,7	7.7	5.8
p-value	P = 0.78	**p = 0.03**	**p<0.001**

N: Number of women for which data were available.

Values in bold indicate statistical significance, as the level of statistical significance was set to p≤0.05.

## Discussion

To our knowledge, this is the first study assessing the impact of social isolation or quarantine on pain intensity, pain perception and on the social support in endometriosis patients. Since the study was performed during the SARS-CoV-2 pandemics, this study reflects a unique perspective of pain and disability perception in a group of German endometriosis patients during imposed social distancing or quarantine. Moreover, the impacts and alterations in HRQoL showed by this study may offer an insight of the possible effects of upcoming public restrictions due to the persistent COVID-19 pandemic or to other socio-environmental changes, which can lead to similar socially burdensome or isolating circumstances in chronic pain patients.

In conformity with the objectives of this study, we assessed the changes in pain characteristics prior to and during social distancing measures. Exacerbation of pain intensity by social distancing measures due to the COVID-19 pandemic was expected by some experts [19]. In contrast, we showed no significant worsening of pain intensity as an early outcome of social distancing. Median menstrual pain significantly decreased after social distancing, whereas no significant changes in pain intensity were observed within the other assessed pain modalities, such as dyspareunia or dyschezia. Nevertheless, when compared to those how experienced decreased pain during the pandemic, more participants described increased pain intensity with respect to everyday pain modalities, such as non-cyclic pain or lower back pain, a fact that may be related to increased pain awareness. Other studies investigating changes in pain intensity in the light of catastrophic or stressful events are in line with our results, as they showed that not all psychological stressors will aggravate chronic pain [20, 23].

Pain is defined not only by intensity but also by other painful experiences, such as functional disability [9]. The pandemic put an additional toll on people with preexisting pain conditions, especially women [24]. Endometriosis related disability is known to influence all aspects of the private and work life [25, 26]. Women, who were in many cases primary in charge with childcare and family responsibility before the pandemic [27], experienced even more burden due to increased time for household chores, childcare and homeschooling during the social distancing measures [28, 29]. These facts may explain the reduced ability of endometriosis patients to recollect from family activities, as shown in this study. In contrast, the global PDI score and specific sub-scores (social, occupational and sexuality) improved during the social distancing period, presumably due to the improvement of menstrual pain, as menstrual pain was described as a predictor of poor performance at work and at home [25].

Pain medication management changed in some participants, as nearly 16% of participants increased the dosage of over-the-counter pain medication and nearly 16% increased the dosage of prescription-only pain medication. Similar developments during the COVID-19 pandemic were published in other chronic pain patients [18]. Because of the design of the study, we cannot distinguish, if these changes were caused by changes in prescription patterns, as described previously [30], or changes in self-medication. Nevertheless, we hypothesize, that these outcomes are partially explained by the attempt to prevent emergency visits, by reduced access of chronic pain patients to high-quality pain management due to closure of pain management services, by cancellation of elective surgeries and by reduced capacities of psychological and interdisciplinary treatment approaches [18, 24, 30, 31].

Pain perception is determined by psychological factors, as well. While the physical impairment caused by pain decreased, pain cognition deteriorated dramatically during social isolation or quarantine. A previous study showed that pain cognition, for example pain awareness and pain anxiety, were worse in women with endometriosis than in healthy controls [32]. This study showed that hypervigilance to pain even increased in 43.6% of the study population during the government-imposed social distancing measures. The increased awareness of pain during the COVID-19 pandemic might be linked to the fear, the symptoms could be a sign of infection with SARS-CoV-2 [19]. Additionally, during social isolation or quarantine, significantly more endometriosis patients experienced pain anxiety, such as feelings of pain as a threat or a bothersome event. Impaired pain cognition was, even to a higher level than pain intensity, related to a lower HRQoL in chronic pain patients [32, 33]. Moreover, impaired pain cognition negatively influenced the acute and long-term effects of medical therapies in chronic pain patients [33–35].

Pain is modulated by social and emotional experience as well [9]. Previous studies showed, that empathy, caring and concern seem to have an overall beneficial effect on pain and sustain the perceived legitimacy of pain complaints [9]. Verbalization of complaints is common in patients with chronic pain, as up to 95.8% talk about their health problems with their social network, the spouse being the most frequently mentioned and most beneficial sharing partner, followed by health care professionals, family and friends [36]. We showed that emotional sharing of pain-related symptoms was significantly altered by the additional acute life event, the COVID-19 pandemic, which affected both, patients and their partner. In this study group, 36.6% stated that they reduced verbalization of pain experience during social isolation or quarantine. This is alarming, as previous studies stated that not being believed might question the legitimacy of the complaints [37]. Additionally, this study showed that during stressful situations such as the COVID-19 pandemic, the closer the relationship was, the higher was the perceived level of support and empathy from significant others, and vice versa, as 14.6%, 21.9% and 31.5% of study participants stated they experienced less support from their partner, family and friends, respectively. It seemed that physical distancing induced emotional and/or psychological distancing. However, these alterations may not correctly reflect the reality, as self-reported social support is only modestly correlated with the actual received support [38]. Additionally, this survey revealed how fast the perceived social support was disrupted in light of altered or new emerged emotional, social and health care constraints relevant to the social network of chronically ill patients. This may have long-term implications for pain patients, as pain intensity and pain interference are negatively affected by social disconnection and loneliness, as caused by the SARS-CoV-2 pandemic, and could lead to exacerbation of pain related disability [24].

The study has several limitations, as participants were acquired with the help of support groups. This fact could lead to selection bias, since women recruited via support groups tended to have more severe symptoms and lower quality of life than those recruited via other methods [39]. However, the study population displayed a good representation of German endometriosis patients, as the median diagnostic delay in our study was 9.62 years, being in line with other studies, which showed a total delay of diagnosis of 10 years in Germany [4]. Nevertheless, we observed a significant drop in the sample size of participants, as 413 accessed the questionnaire, but only 285 answered at least one of the questions regarding pain intensity, pain induced disability or emotional and social pain experience. We did not notice significant differences in assessed clinical and demographic characteristics between those how did or did not respond to the questions, except for the duration of social isolation or quarantine. Further, the study participants were asked to recall their pain and disability level during the four weeks prior to the government-imposed lockdown. Recall is always susceptible to some bias. However, recall is a common tool in the field of endometriosis, as diagnosis and treatment planning are guided substantially by retrospective pain assessment [40]. Previous findings showed that women with endometriosis were relatively accurate in their recall of pain [40]. Nevertheless, no studies assessed the accuracy of recall regarding pain induced disability. Moreover, as the survey was conducted during the first wave of the SARS-CoV-2 pandemic, we do not know, if the responses accurately depict the current state of the pandemic, as adaptive behaviors may have occurred over time.

Despite some limitations, this study summarize and raise awareness regarding important patient-related outcomes in women suffering from endometriosis, supporting the notion, that an acute stressful event on national or international scale might easily disrupt the social support network, resulting in a negative impact on perceived social and emotional support in this collective. Thus, we suggest that society and health care systems should beforehand prepare to provide medical and social safety nets with respect to challenging times, which should be accessible easily and without obstacles. Moreover, clinicians should encourage patients to join support groups and they should advise them to share their pain-related symptoms with their social network. Additionally, it would be helpful, that health care systems provide advice for the general population, how to deal with chronically ill patients, especially in difficult times.

Further analyses are crucial to elucidate the complex relationship between patients with chronic pain and their social network and how changes in social interactions might influence the short-term and long-term physical and psychological well-being. Further studies are needed to assess factors which may help to empower women with chronic pain, especially in challenging situations.

## Supporting information

S1 File Web-based questionnaire. (DOCX)
