# Supplementary material for: Pain experience and social support of endometriosis patients during the COVID-19 pandemic in Germany – results of a web-based cross-sectional survey
Source: PLoS One. 2021 Aug 25;16(8):e0256433. doi: 10.1371/journal.pone.0256433 (PMC8386836; doi:10.1371/journal.pone.0256433)
Supplement: S1 File — (DOCX) [file pone.0256433.s001.docx]

**Web-based questionnaire**

1. How old are you? ________years
2. Do you live alone?

- No
- I live with my partner/family
- No, I live in a shred accommodation
- Others: __________________

1. Do you experience quarantine or do you reduce your social relationships (social distancing)?

(Quarantine is a governmental or a self-imposed isolation with the aim to protect others from becoming infected. Social isolation is avoiding direct physical social contacts for disruption of infection chains.)

- No
- I am reducing social contacts
- I am in quarantine

1. For how long did you adhere to social distancing or did you experience quarantine? _____days
2. Do you have a stable relationship?

- Yes
- No

1. How much did you reduce your social contacts?

- Not at all
- Quite a bit
- Moderate
- Considerable
- Significantly

1. For how long do you have an endometriosis diagnosis? ________years
2. Since when do you have pain (cyclic or non-cyclic pain, pain at intercourse, pain at urination or defecation)? __________years
3. How would you describe your pain?

- Continuous pain with small fluctuations
- Continuous pain with considerable fluctuations
- Pain attacks, no pain in between
- Pain attacks, pain in between

1. How did you usually experience your pain levels during the time period four weeks previous to social isolation or quarantine (continuous pain scale/slide bar 0-100)?

Cyclic pain

Non-cyclic pain

Pain at intercourse

Pain during urination

Pain during defecation

Lower back pain

1. Please mention the degree to which level several aspects of your life were disrupted by pain during the last four weeks before the implementation of social isolation or quarantine. We would like to know how much pain is preventing you from leading a normal life?

For each of the seven categories of life activity listed, please circle the number on the scale which describes the level of disability you typically experience. A score of 0 means no disability at all, and a score of 10 signifies that all of the activities in which you would normally be involved have been totally disrupted or prevented by your pain.

1. Family/Home Responsibilities. This category refers to activities related to the home or family. It includes chores and duties performed around the house (e.g. yard work)
2. Recreation. This category includes hobbies, sports and other similar leisure time activities.
3. Social Activity. This category refers to activities which involve participation with friends and acquaintances. It includes parties, theaters, concerts, dining out and other social functions.
4. Occupation. This category refers to activities that are a part of or directly related to one`s job. This includes nonpaying jobs as well, such that of a housewife.
5. Sexual behavior. This category refers to the frequency and quality of one`s sex life.
6. Self-Care. This category includes activities which involve personal maintenance and independent daily living (e.g. taking a shower, driving, getting dressed, without getting foreign help).
7. Life-Support Activity. This category refers to basic life-supporting behaviors such as eating, sleeping, and breathing.
8. How did you usually experience your pain levels during the time period since the implementation of social isolation or quarantine (continuous pain scale/slide bar 0-100)?

Cyclic pain

Non-cyclic pain

Pain at intercourse

Pain during urination

Pain during defecation

Lower back pain

Please mention the degree to which level several aspects of your life were during the last four weeks before the implementation of social isolation or quarantine were disrupted by pain. We would like to know how much pain is preventing you from leading a normal life?

1. Please mention the degree to which level several aspects of your life were disrupted by pain since the implementation of social isolation or quarantine. We would like to know how much pain is preventing you from leading a normal life?

For each of the seven categories of life activity listed, please circle the number on the scale which describes the level of disability you typically experience. A score of 0 means no disability at all, and a score of 10 signifies that all of the activities in which you would normally be involved have been totally disrupted or prevented by your pain.

1. Family/Home Responsibilities. This category refers to activities related to the home or family. It includes chores and duties performed around the house (e.g. yard work)
2. Recreation. This category includes hobbies, sports and other similar leisure time activities.
3. Social Activity. This category refers to activities which involve participation with friends and acquaintances. It includes parties, theaters, concerts, dining out and other social functions.
4. Occupation. This category refers to activities that are a part of or directly related to one`s job. This includes nonpaying jobs as well, such that of a housewife.
5. Sexual behavior. This category refers to the frequency and quality of one`s sex life.
6. Self-Care. This category includes activities which involve personal maintenance and independent daily living (e.g. taking a shower, driving, getting dressed, without getting foreign help).
7. Life-Support Activity. This category refers to basic life-supporting behaviors such as eating, sleeping, and breathing.
8. How do you perceive the pain in comparison to the pain experience before the implementation of social isolation or quarantine? (five-point Likert scale: significantly diminished/less, less, neutral, more, significantly more)
9. I am experiencing as much stress because of the pain as before the implementation of social isolation or quarantine.
10. I am able to relax as good as before the implementation of social isolation or quarantine.
11. I am experiencing the pain as frequently as before the implementation of social isolation or quarantine.
12. I am able to cope with the pain as good as before the implementation of social isolation or quarantine.
13. I am experiencing the pain as disturbing as before the implementation of the social distancing or quarantine.
14. I am experiencing the threat of pain equal as before the implementation of social distancing or quarantine.
15. I am taking as many over the counter pain medication as before the implementation of social distancing or quarantine.
16. I am taking as many prescriptions only pain medication as before the implementation of social distancing or quarantine.
17. I am addressing the pain issue as often as before the implementation of social distancing or quarantine.
18. How do you experience the support of your partner, family or your circle of friends? (five-point Likert scale: significantly diminished/less, less, neutral/not changed, more, significantly more)
19. My partner is supporting me during the pain just as well as before the implementation of social distancing or quarantine.
20. My partner is taken my pain as seriously as before the implementation of social distancing or quarantine.
21. My family is supporting me during the pain just as well as before the implementation of social distancing or quarantine.
22. My family takes my pain as seriously as before the implementation of social distancing or quarantine.
23. My circle of friends is supporting me during the pain just as well as before the implementation of social distancing or quarantine.
24. My circle of friends is taking my pain as seriously as before the implementation of social distancing or quarantine.
